# High Levels of Community Support for Mansonellosis Interventions in an Endemic Area of the Brazilian Amazon

**DOI:** 10.3390/tropicalmed10070186

**Published:** 2025-07-02

**Authors:** Uziel Ferreira Suwa, Carla Letícia Gomes Simão, Ulysses Carvalho Barbosa, Patrícia Moura Sousa, Cláudia Patrícia Mendes de Araújo, Marilaine Martins, James Lee Crainey

**Affiliations:** 1 Laboratório de Ecologia de Doenças Transmissíveis na Amazônia, Instituto Leônidas e Maria Deane, Fundação Oswaldo Cruz Amazônia, Manaus 69057-070, Amazonas, Brazil; usuwa@fiocruzbr.onmicrosoft.com (U.F.S.); carla-simao@live.com (C.L.G.S.); pmsousa@fiocruzbr.onmicrosoft.com (P.M.S.); claudiacrainey@gmail.com (C.P.M.d.A.); 2 Programa de Doutorado em Saúde Pública na Amazônia, Instituto Leônidas e Maria Deane, Manaus 69057-070, Amazonas, Brazil; 3 Programa de Mestrado em Condições de Vida e Situações de Saúde na Amazônia, Instituto Leônidas e Maria Deane, Manaus 69057-070, Amazonas, Brazil; 4 Laboratório de Etnoepidemiologia, Coordenação de Sociedade Ambiente e Saúde, Instituto Nacional de Pesquisas da Amazônia, Manaus 69067-375, Amazonas, Brazil; barbosaulysses06@gmail.com; 5 Programa de Doutorado em Biologia da Interação Patógeno Hospedeiro, Instituto Leônidas e Maria Deane, Manaus 69057-070, Amazonas, Brazil; 6 Fundação de Medicina Tropical Dr. Heitor Vieira Dourado, Manaus 69040-000, Amazonas, Brazil

**Keywords:** mansonellosis, *M. ozzardi*, *M. perstans*, mass drug administration, Amazonas

## Abstract

Mansonellosis is a chronic infectious tropical disease that affects hundreds of millions of people worldwide but is not currently targeted for control. In this study, we interviewed 320 residents from Sao Gabriel do Cachoeira (SGC) about their support for soil transmitted helminth (STH) and mansonellosis interventions. Our survey found no significant difference between community support for mansonellosis and STH disease treatment when comparing any equivalent treatment regimen or program, although support for STH treatments was always higher than for mansonellosis treatments. No significant differences were detected when comparing community members’ willingness to participate in treatment programs and their willingness to allow family members to participate in an equivalent program. Our survey did, however, almost always find that significantly more community members were willing to participate in a proposed treatment program if the treatment regimen of that program was shorter than an otherwise equivalent regimen. Although significantly fewer people said they would participate in a curative four-week treatment course for mansonellosis than in a mansonellosis mass drug administration (MDA) program, significantly more community members said they would take a curative mansonellosis treatment course that lasted seven days or less than they would participate in any type of anthelminthic MDA proposed to them. The number of community members who said they would participate in any helminthic treatment program if they knew there was a ≥50% chance that they were infected was significantly higher than the number who said that they would without knowing the regional prevalence rate.

## 1. Introduction

Mansonellosis is a chronic human filarial disease caused by filarial parasites belonging to the genus *Mansonella* [1]. In the Americas, the disease is caused by *Mansonella ozzardi* and *Mansonella perstans* with *M. ozzardi* mono-infections predominating in all but the most northwestern regions of the Brazilian Amazon, where *M. perstans*, *M. perstans*, and *M. ozzardi* co-infections are known to occur [2,3,4]. It has been estimated that there are around 12 million chronic mansonellosis infections in the Amazon region, with prevalences varying greatly from close to zero in some urban areas, like Manaus, to greater than 50% in some heavily forested indigenous regions [4,5,6,7]. Although there is growing concern about the hitherto uncalculated disease burden of mansonellosis and an increasing number of specialists are calling for infections to be treated, there are still no national or regional treatment guidelines for mansonellosis in Brazil or in any other country where the disease is endemic [4,8,9].

Like many human filarial parasite infections, mansonellosis infections caused by *M. perstans* can be effectively cured with a course of doxycycline, which targets the filarial parasite *Wolbachia* endosymbiont [10,11]. It is also likely that *Wolbachia*-targeting treatments could be used for *M. ozzardi* infections and *M. ozzardi* and *M. perstans* co-infections [4,8]. Existing *Wolbachia*-targeting treatments, such as doxycycline, require long treatment courses (of between 4 and 6 weeks) to be effective and thus are not yet widely used in filarial disease control or elimination programs [8,12,13]. Novel fast-acting *Wolbachia* treatments, as well as other curative macrofilaricidal treatments are being developed for onchocerciasis and lymphatic filariasis programs and are likely to be effective for mansonellosis control programs [4,9,12,13,14]. Such treatments could radically improve the feasibility of controlling numerous filarial diseases, including mansonellosis, and could also be used in synergy with traditional anti-helminthic chemotherapeutics, such as ivermectin [4,9,12,13,14].

While the WHO’s onchocerciasis and lymphatic filariasis elimination plans are likely to increasingly deploy macrofilaricidal chemotherapeutics, for the near and medium-term future, their programs are likely to still be principally focused on the use of traditional anti-helminthic microfilarial drugs which aim to reduce morbidity by breaking filarial parasite transmission [15,16,17]. Many of the microfilaricidal drugs currently used by the WHO in their mass drug administration programs (MDAs) are also effective against parasites that cause mansonellosis and thus could be used in a similar way to break the transmission of mansonellosis [4,8,12,14]. Some also have utility in the treatment of other helminthic diseases, such as soil-transmitted helminth (STH) infections [14,18,19,20,21]. Ivermectin, a highly effective microfilaricidal treatment against *M. ozzardi*, *Onchocerca volvulus,* and *Wuchereria bancrofti*, is also effective against *Ascaris lumbricoides*, *Trichuris trichiura,* and *Strongyloides stercoralis* [18]. And while ivermectin is not effective in clearing *M. perstans* microfilariae, *M. perstans* microfilariae can be treated with benzimidazoles (like mebendazole and albendazole), which are widely used as STH treatments [14,18,19,20,21]. Although the optimal treatment and dosage regimens for helminthic infections are not the same for all helminthic parasite infections, there is still considerable scope for disease control planning synergies in helminthic disease control programs [12,13,18,19,20,21].

A key concern regarding the viability of MDAs for filarial diseases, including mansonellosis, is community participation. Analysis of onchocerciasis and lymphatic filarial programs suggests that ivermectin-based MDA control programs cannot be expected to operate efficiently if they do not have ≥65% coverage [22,23,24,25,26]. Although it is not clear how long *M. ozzardi* adults live naturally, it is likely that both *M. ozzardi* and onchocerciasis foci require similar ivermectin program lengths for elimination and that they can be similarly enhanced by increasing community participation [1,4,27,28]. However, there is almost no information available on community support for any kind of mansonellosis disease control program anywhere in the world. Data on this for the Brazilian Amazon is particularly valuable as mansonellosis in the Amazon region is caused predominantly by *M. ozzardi*, which can be effectively cleared by ivermectin, meaning that MDA is expected to be very effective at breaking mansonellosis transmission in the Brazilian Amazon region [4,14,27,28,29,30]. Given this and recent advances in macrofilaricidal drug development which mean that a suite of fast-acting and potentially curative treatments are likely to soon be available for mansonellosis treatment and control, this work aimed to collect baseline data that can not only evaluate the viability of different types of disease control interventions but also provide valuable information about how any such programs might be optimized.

## 2. Materials and Methods

### 2.1. The Study Site

**Figure 1:**
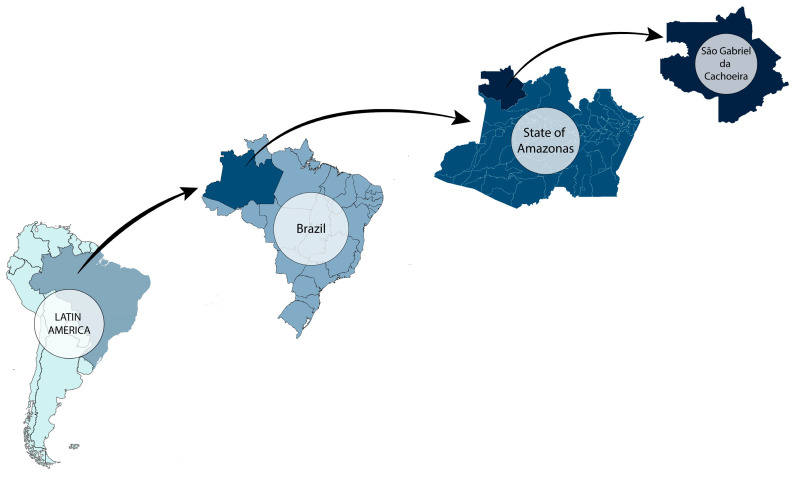
A map of South America highlighting the locality of Sao Garbiel do Cachoeira. The figure is composed of four plates: Plate 1 highlights the boarders of Brazil within the continent of South America; Plate 2 highlights the Brazilian state of Amazonas within the borders of Brazil; Plate 3 highlights the municipality of SGC within the borders of the Brazilian state of Amazonas; Plate 4 shows the borders of the municipality of SGC.

**Figure 2:**
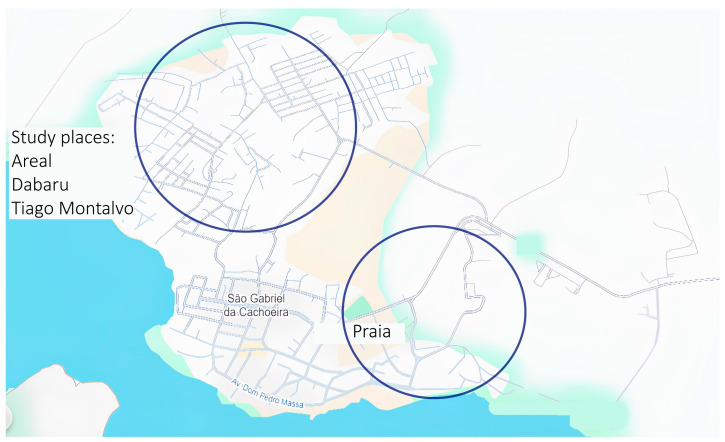
A street map of urban Sao Garbiel da Cachoeira. A street map of urban SGC adapted from PM/SGC 2023. Blue circles are used to highlight the neighborhoods targeted for our survey.

The study was conducted in an urban area of the Brazilian municipality, São Gabriel da Cachoeira (SGC), which is located in the extreme northwest region of Brazil’s “Amazonas state” (see Figure 1 and Figure 2). The municipality is 852 km from Manaus, the state capital, and is situated on the banks of the Rio Negro, close to the Brazilian border with Colombia and Venezuela. SGC is one of the largest municipalities in Brazil; however, with a 2022 population measurement of 51,795 inhabitants it is also one of the least densely populated. The Human Development Index (HDI) of SGC has been calculated as 0.609, and approximately 90% of the SGC population identify as indigenous (Instituto Brasileiro de Geografia e Estatística [IBGE], 2022) [31]. The study site was chosen because it is endemic for mansonellosis and STHs [2,3,32,33,34]. The study area included neighborhoods that have been used in several previous epidemiological studies on mansonellosis: Areal, Tiago Montalvo, Dabaru, and Praia (Figure 1 and Figure 2) [2,3,34,35].

### 2.2. Data Collection

A questionnaire enquiring about community acceptance of anthelmintic drug treatments and MDA was drafted and submitted to an ethics committee together with two complementary helminth parasite fact sheets that were intended to be provided to all community members invited to participate in the study. Following minor revisions, the project proposal, questionnaire (Figure S1), informed consent form (Figure S2), and two fact sheets (Figures S3 and S4) used for this study were approved by the “Comitê de Ética em Pesquisa da Fundação de Medicina Tropical Doutor Heitor Vieira Dourado” (CEP/FMT-HVD) and given the protocol number: 73732523.7.0000.0005. Four trained researchers then interviewed 320 residents of SGC in their homes using the ethically approved questionnaire. Following the interviews, the researchers offered two helminth fact sheets to all the residents who were approached, independently of whether they agreed to participate in the study. The questionnaire was administered daily between 8:00 am and 5:00 pm by teams of four researchers with the authorization and assistance of Community Health Agents (CHAs) who worked in the Basic Health Units (UBS) of the neighborhoods under study. Each researcher interviewed one eligible resident per household (randomly selected from the household). The questionnaire was divided into four blocks, and information was collected on four themes: (1) socioeconomic and demographic conditions, (2) knowledge about common helminthic diseases in the region and their control methods, (3) experiences with common helminthic diseases in the region, and (4) attitudes toward treatments for common helminthic diseases in the region. The work presented here focuses on the data provided in Section 1 and Section 4 of the questionnaire.

### 2.3. Eligibility Criteria

This study included only adults aged ≥ 18 years living in the urban area of São Gabriel da Cachoeira. Individuals who presented with deficits in cognitive function, especially memory loss, or cognitive incapacity due to mental illness were excluded from the study.

### 2.4. Data Analysis

Initially, the information obtained was subjected to descriptive statistical analysis, expressed as absolute numbers and percentage indices. Bivariate analysis was performed using Pearson’s chi-squared test (χ^2^). In all statistical analyses, statistical significance was set at *p* < 0.05. Statistical tests were performed using the online tool https://www.socscistatistics.com/tests/chisquare/default2.aspx (accessed on 25 May 2025).

## 3. Results

### 3.1. Sociodemographic Profile of Surveyed SGC Residents

Our sociodemographic survey showed that volunteers who agreed to participate in our survey were typical of the population of Sao Gabriel do Cachoeira and the rural areas of Amazonas state, where mansonellosis is endemic. Table 1 and Table 2 provide a detailed account of the key demographic features of the surveyed population. Of the 320 individuals included in our study, 105/320 (32.8%) were male and 215/320 (67.2%) were female. The interviewees were residents of the following neighborhoods: Praia 113/320 (35.3%), Tiago Montalvo 88/320 (27.5%), Areal 82/320 (25.6%), and Dabaru 37/320 (11.6%). The mean age of participants was 40.8. A total of 294/320 (91.9%) self-identified as indigenous; 134/320 (41.9%) reported being single and 252/320 (78.8%) had ten or more years of schooling (Table 1). The most frequently reported occupation categories were civil servant 64/320 (20%), farmer 59/320 (18.4%), and homemaker 56/320 (17.5%). A total of 276/320 (86.3%) interviewees had a family income equivalent to less than three minimum wages. The average number of residents per household was 5.5 and 162/320 (50.6%) had lived at the address where they were interviewed for more than 10 years. Using the Brazilian Association of Research Companies (ABEP) (2022) classification system, 258/320 (80.6%) interviewees belonged to classes C2 or DE, of which 190/320 (59.3%) were classified as DE, and 68/320 (21.3%) were in class C2 (see Table 1 and Table 2).

### 3.2. SGC Community Support for Mansonellosis and SHT Treatment Programs

Our survey was unable to detect significant differences in the number of people who said they would participate in any given treatment regimen and the number of people who said they would allow a family member to participate in an equivalent regimen. Thus, our survey found that if helminth treatment or control programs were implemented in SGC, the level of adult participation and the level of participation of minors (based on guardian consent for their participation) are unlikely to be significantly different for any kind of helminthic medication treatment or treatment regimen, and that high levels of adolescent and child participation can be expected (Table 3 and Table 4). Similarly, our study also found no significant differences between the number of participants who said they would participate in an STH treatment program or allow a family member to participate in an STH treatment program, and the number of people who said that they would participate or allow a family member to participate in an equivalent mansonellosis treatment program (see Table 3, Table 4, Table 5 and Table 6). Although our survey consistently detected lower levels of support for mansonellosis treatment regimens, this support was never significantly lower, suggesting that the type of helminthic infection a community member had did not have a major impact on their willingness to participate in a treatment program (Table 3, Table 4, Table 5 and Table 6).

**Figure 3:**
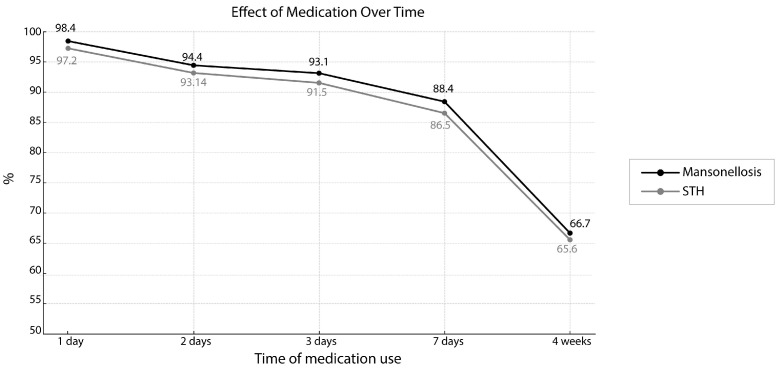
Community support for therapeutic regimens of varying durations. The proportion (as a percentage) of the community of SGC whom said they would participate in either a STH or mansonellosis treatment is plotted on the *Y* axis of this graph. The duration of a proposed treatment course is plotted on the *X* axis. Data for response concerning STH infections is shown with a black line; data concerning mansonellosis infections is shown with a gray line.

Our study, however, found strong evidence that increasing the helminth treatment course length had a significant effect on the number of people who would participate. Significantly more community members said that they were prepared to take a single dose of treatment, or allow a family member to take a single dose of treatment (for an STH or mansonellosis infection), than would take any type of longer treatment for themselves or a family member (see Table 3, Table 4, Table 5 and Table 6). Longer proposed treatment courses always had fewer community members willing to participate than shorter equivalent treatment courses (see Figure 3 and Table 3, Table 4, Table 5 and Table 6). Comparing between any two equivalent treatment regimens for either STH or mansonellosis infections, we almost always found significantly higher numbers prepared to take the shorter treatment regimen (see Table 3, Table 4, Table 5 and Table 6). The only STH treatment regimen comparison that did not have significantly fewer people saying they were willing to participate in the longer treatment course was the comparison made between the 2-day and 3-day treatment courses (Table 3 and Table 4). Comparisons between community support for 2- and 3-day mansonellosis treatment courses were also not significantly different (Table 3 and Table 4). And although we found significantly more SGC community members willing to take a 3-day rather than a 7-day mansonellosis treatment course for themselves, the number of people willing to allow a loved one take a 3-day treatment course (compared to a 7-day treatment) was not significantly higher (see Table 4 and Table 6). All other comparisons made between otherwise equivalent long and short treatment courses were, however, significant (see Table 3, Table 4, Table 5 and Table 6).

Importantly, our study also found that SGC community members were much more likely to agree to a treatment program if they knew that they had a helminthic disease than if there was any uncertainty about whether they were infected (see Table 5 and Table 6). Almost all the community members said that they would take helminthic disease treatment (for either an STH or mansonellosis infection) if they were sure they were infected, and significantly more said they would participate in MDA without knowing if they were infected, provided they knew there was a ≥50% chance they were infected than if they simply knew the disease was endemic in the area (see Table 5 and Table 6).

## 4. Discussion

### 4.1. High Levels of Community Member Willingness to Participate in Anthelmintic Disease Programs Suggests Such Programs Could Work Efficiently in SGC

Our survey found that the proportion of the SGC community that said they would participate in any given treatment regimen was broadly in line with the levels of community participation typically observed in African helminthic disease control programs. Recent studies on community participation in STH MDAs in Malawi and Benin have reported that 73–100% of the community agreed to participate in anti-helminth MDAs [36,37,38]. A total of 72.5% of our participants said they would participate in an anti-helminthic MDA and 71.6% said they would allow a family member to participate in a STH MDA. Almost all (98.4%) of the community said they would take a single dose of an anti-helminthic treatment if they knew they were infected; therefore, the number of SGC community members who said they were willing to participate in single-dose SHT treatment programs can be considered to be between 71.6% and 98.4% (depending on the regional prevalence levels) and, thus, the expected coverage of an STH MDA control program in SGC and the wider Amazon could be expected to be broadly similar to what is observed in Africa.

Given that mansonellosis infections are often regarded as benign and do not have a recognized disease burden, unlike many helminthic diseases have, we were concerned that low levels of community support for mansonellosis control programs could render certain types of control programs inviable. Slightly fewer survey participants said they would allow their family members to participate in an MDA for mansonellosis than said they would allow themselves to participate in any given anti-helminthic MDA (see Table 3, Table 4, Table 5 and Table 6); however, even so, it appears from our survey that coverage for an MDA mansonellosis program implemented in the Brazilian Amazon is likely to be similar or even slightly higher than what is observed in the WHO’s African filarial programs [39,40,41,42]. Most of the WHO’s onchocerciasis and lymphatic filariasis disease control programs in West Africa, which have significantly reduced the disease burden of both diseases and eliminated new infections in many foci, have used an MDA approach, with participants receiving a periodic (annual or biannual) single dose of ivermectin (often referred to as Community-Directed Treatment with ivermectin [CDTi]) [42,43,44]. As the same ivermectin doses used in these programs can effectively eliminate *M. ozzardi*, and *M. ozzardi* is responsible for the vast majority of mansonellosis infections in Amazonas state, a mansonellosis control program modeled on West-African CDTi programs could reasonably be expected to have similar effects, provided that participation levels were comparable [4,14,29,30]. Typically, the WHO’s CDTi programs report between 60 and 70% of eligible members of the community participating [23,24,25]. As our survey found that between 72.2% and 79.4% of respondents would participate in a CDTi program for mansonellosis, depending on whether or not they knew the local level of parasite prevalence; our survey suggested that ivermectin-based control could be viable in SGC. Of course, saying that one would participate in a study is not the same as actually participating and, thus, it is possible that our survey could be overestimating the actual number of people willing to participate in a program. A previous research study, however, found that 100% (85/85) of patients who were told they were infected with *M. ozzardi* parasites and were supplied with a single dose of ivermectin, took that treatment. This observation is in line with the data collected from our questionnaire, which suggested that 97.2% of the population would take the treatment if they knew they were infected. Collectively, this suggests that what SGC community members say they will do, regarding anti-helminthic treatments, and what they actually do, is probably very similar [45].

### 4.2. Community Participation in Mansonellosis and STH Treatment Programs Can Likely Be Enhanced with Fast-Acting Drug Treatments

Although most mansonellosis cases in the Amazon region are caused by *M. ozzardi* infections and could be targeted with a CDTi approach, mansonellosis in SGC is also caused by *M. perstans, M. ozzardi,* and *M. perstans* co-infections [2,3,4]. Similar to many SHT treatments, *M. perstans* treatment requires medicinal courses that last for several days or weeks. Clearing *M. perstans* microfilariae from the blood requires at least two weeks of treatment with mebendazole, and curative treatment requires six weeks of doxycycline [8,9,10,11,12,13]. New potentially curative anti-filarial drugs (such as Oxfendazole and Rifampicin), which have short treatment times of a week or less, are currently in clinical trials and could soon become available for all types of mansonellosis infections [4,5,9,10,46]. These new treatments could radically expand the treatment and control options for mansonellosis infections caused by *M. perstans* and some could potentially be used for *M. ozzardi* infections too [4,8,12,13]. There are also many new macrofilaricidal and potentially curative treatments in various stages of development which are, in the near future, likely to make all filarial disease control programs, including mansonellosis control programs, much more financially and logistically viable [4,8,12,13,14]. It is important for future control programs to have data on how such drugs could influence participation so that they can be optimized [8,14]. Our study found that significantly more community members said they were prepared to take a single dose of treatment (to treat an STH or mansonellosis infection) than would take a longer treatment and that the longer the treatment course proposed was, the fewer participants there were that were willing to say they would participate (see Figure 3 and Table 3 and Table 4). Interestingly too, our survey found that treatment duration was a more important consideration for the SGC community when determining whether they would participate in a treatment course than the type of helminthic infection they had (see Table 3, Table 4, Table 5 and Table 6). While we did not detect a significant difference between the number of people who said they would take a treatment course for an SHT infection and mansonellosis infection for any of the five treatment courses that we proposed, we found that the only treatment course extension that did not have significantly fewer people saying they would agree to participate was a change between two and three days of treatment (see Table 3 and Table 4). Our results, thus, highlight the desirability of new fast-acting drugs for future cost-effective and efficient anti-helminthic disease control programs.

### 4.3. Community Participation in Mansonellosis and STH Treatment Programs Can Likely Be Enhanced with Diagnostic Testing and Prevalence Surveys

Our results showed that significantly more people said that they would participate in an MDA if they knew that they were more likely than not to be infected with a mansonellosis parasite. Only 70% of the community said that they would participate in an MDA like those used by the WHO for filarial disease control in Africa if they knew the region was endemic, but they did not know the regional prevalence rates. However, almost 80% said that they would participate if they knew infection rates were ≥50%, and almost all said that they would participate if they knew they were infected. Although community participation rates for CDTi programs are typically lower than 80%, and effective control and elimination have been achieved with lower coverage rates, 80% coverage is the target for most filarial control programs. From our survey, it appears that, at least in highly endemic areas (with prevalence rates ≥50%), this goal could potentially be met for a CDTi program targeting mansonellosis if baseline community prevalence surveys were conducted prior to the implementation of a program. Without clinical features such as nodules, mansonellosis prevalence estimates need to rely on blood surveys [1]. As previous mansonellosis epidemiological studies have shown that large numbers of *M. ozzardi* infections go undetected by traditional blood smear and light microscopy surveys, it would be important to ensure that, if surveys were not conducted directly with PCR assays, survey prevalence estimates took this fact into account in the same way that WHO onchocerciasis prevalence estimates from nodule surveys take into account the fact that many people infected with *O. volvulus* do not have visible nodules [47].

Currently, the WHO uses doxycycline and a test-and-treat (TTd) strategy for onchocerciasis control in a limited number of regions of West Africa where it cannot use its ivermectin-only strategy safely [48]. This approach could also be employed to treat and cure mansonellosis infections in SGC, which cannot be effectively targeted using an ivermectin-only MDA approach [4,8,14]. However, doxycycline filarial treatments require 4- to 6-week therapies [12,13]. Despite being potentially curative for many filariasis infections, TTd strategies sometimes report lower levels of participation than is reported for CDTi programs. A recent onchocerciasis TTd strategy in Africa, for example, found that only 66% agreed to participate in the program [32,34]. While these low levels of participation are very likely to be at least partially explained by the current necessity for painful skin snips (biopsies) to be taken for the diagnostic component of these strategies, our survey suggests that long treatment times might also have an important impact on community participation. Interestingly, our study found that shorter treatment courses could significantly increase community participation in either a STH or mansonellosis program. Our survey found that a 4-week test-and-treat (TT) program (using an unspecified drug) for mansonellosis would have nearly identical levels of participation (65.5%) to those observed for onchocerciasis TTd programs in Africa, but that participation could be expected to increase significantly to 86.5% if the treatment course could be shorted to seven days; 91.5% if the treatment course could be shortened to three days; 97.2% if the treatment could be given in a single dose [8,14]. Two short-course and potentially curative drug treatments, which use Oxfendazole and Rifampicin, are currently in clinical trials [8,12,13]. Both these treatments could potentially be used for mansonellosis infections and are likely to have treatment courses shorter than 7 days [8,12,13]. Mansonellosis TT programs using these new drugs or other new shorter-course drugs could, thus, reasonably be expected not only to have lower levels of treatment dropout but also to have significantly higher levels of community participation than are currently observed in both standard onchocerciasis CDTi programs and TNT programs. Unlike Oxfendazole, Rifampicin, is not specifically being tested against *M. perstans*; however, the fact that doxycycline is effective against *M. perstans* and *M. ozzardi* harboring *Wolbachia* strongly suggests that it, and most *Wolbachia*-targeting therapies, will be effective against all forms of mansonellosis [8,12,13]. As a front line drug against tuberculosis, there may be concerns about using Rifampicin to control mansonellosis in the amazon region; however, many other *Wolbachia*-killing compounds with the potential to be developed for short-course anti-filarial treatments, some which are expected to have treatment courses as short as 3 days or less, have been identified and are being assessed for their utility to filarial disease control [8,12,13,48]. Our study, thus, suggests that mansonellosis TT programs that use drugs with a treatment course of three days or less could be expected to have >91.5% community participation, and thus that such drugs could radically change the viability of mansonellosis control in the Brazilian Amazon and beyond. These drugs coupled with new AI-supported diagnostic tools could, therefore, greatly enhance the viability of regional TT programs and thus mansonellosis control in the Amazon and beyond [49,50].

## 5. Conclusions

This study provides baseline data showing that while support for STH treatment and control programs is higher than for equivalent mansonellosis treatment and control programs, the levels of support for STH programs are not significantly higher and that both types of programs have high levels of community support. Our results suggest that a range of mansonellosis treatment strategies may be viable for disease control in the Brazilian Amazon region and beyond. Our study also shows that mansonellosis CDTi programs could be expected to get very close to the standard 80% coverage target in highly endemic areas, provided that treatment programs are accompanied by prevalence surveys. Additionally, our survey showed that mansonellosis TT strategies could expect similar levels of community participation to those used for onchocerciasis in Africa, and importantly, that significantly higher levels of participation could be expected if new short-course treatments became available. Our survey suggests that mansonellosis TT strategies could achieve coverage levels as high as 97.2% for a single-dose drug and levels above 91.5% for all treatment courses of a week or less. In summary, our survey suggests that new short-course curative anti-filarial drugs and TT strategies used in dedicated mansonellosis programs or in combination with traditional drugs such as ivermectin or in synergistic helminthic disease control programs could radically improve the viability of mansonellosis control in the Brazilian Amazon.

## Figures and Tables

**Table 1:** The demographic profile of SGC community members that participated in our survey.

Variables	*n*	(%)
**Sex**		
Male	105	32.8
Female	215	67.2
**Age (years)**		
18–28	88	27.5
29–39	85	26.6
40–50	60	18.8
51–60	45	14.1
>60	42	13.1
**Race/Skin Color**		
White	7	2.2
Black	2	0.6
Brown	17	5.3
Indigenous	294	91.9
**Marital status**		
Single	134	41.9
Married	100	31.3
Widower	17	5.3
Divorced	6	1.9
Others	63	19.7
**Level of education (years)**		
Between 0 and 9	68	21.25
≥10	252	78.8

**Table 2:** The socio-economic and socio-demographic profile of the SGC community members that participated in our survey. The socio-economic classifications used in this table were devised by Brazilian Association of Research Companies (ABEP). Households with A, B1, and B2 ABEP classifications are affluent and have high and upper-middle incomes. Households with a C1 classification are considered as belonging to an emergent middle class and to have moderate spending power. Households with C2 classifications are low income and lower middle class. Households with D-E classifications can be described as living in the least affluent conditions of Brazil and include very low income and poverty-stricken families.

Variables	*n*	(%)
**Work/occupation**		
Farmer	59	18.4
Civil servant	64	20.0
Housewife	56	17.5
Student	10	3.1
Retiree	30	9.4
Unemployed	40	12.5
Other services	61	19.1
**Family income**		
Less than 3 minimum wages	276	86.3
≥3 minimum wages	44	13.8
**Nº Number of residents in the household**		
Up to 4 people	128	40.0
5 or more people	192	60.0
**Time spent at the address**		
0–5 years	98	30.6
6–10 years	60	18.8
>10 years	162	50.6
**Neighborhood**		
Areal	82	25.6
Dabaru	37	11.6
Praia	113	35.3
Tiago Montalvo	88	27.5
**Economic situation**		
A or B1 or B2 or C1	62	19.4
C2 or D-E	258	80.6

**Table 3:** Community support for different lengths of helminthic treatment regimens.

STH					Mansonellosis				
	Self		Family Member			Self		Family Member	
	N	%	N	%		N	%	N	%
1 day	315	98.4	314	98.1	1 day	311	97.2	308	96.25
2 days	302	94.4	302	94.3	2 days	298	93.14	294	91.8
3 days	298	93.1	295	92.1	3 days	293	91.5	289	90.3
7 days	283	88.4	280	87.5	7 days	277	86.5	278	86.8
4 weeks	210	66.7	206	64.3	4 weeks	204	65.6	206	64.3

This table shows the number of SGC community members (as an absolute and as a proportion of study group [*n* = 320]) who said they would take anti-helminthic treatment or allow a family member they are responsible for to receive a treatment for mansonellosis or STH infections. Data for treatment courses of 1 day, 2 days, 3, days, 1 week, and 4 weeks are shown.

**Table 4:** Significant differences in community support for different durations of helminthic treatment regimens in SGC.

	STH		Mansonellosis	
Treatment Regimenn	*p*-Value	*p*-Value (Family Member)	*p*-Value	*p*-Value (Family Member)
1 Day vs. 2 Days	0.005767	0.012534	0.016686	0.019197
3 Days vs. 7 Days	0.040404	0.049662	0.042725	0.171368
7 Days vs. 4 Weeks	0.00001	0.00001	0.00001	0.00001

This table highlights significant differences in community support for helminth infection treatment regimens of differing durations. Quoted *p*-values were obtained from chi-squared tests that compared the number of SGC community members who said they would participate in a given treatment regimen of a stated duration with the number of SGC community members that said they would participate in an equivalent treatment regimen of a different duration.

**Table 5:** Participation in mass drug administration (MDA) programs against helminthiasis among individuals in São Gabriel da Cachoeira-AM.

STH					Mansonellosis				
	Self		Family Member			Self		Family Member	
	N	%	N	%		N	%	N	%
Without prior diagnosis of contamination	232	72.5	229	71.6	Without prior diagnosis of contamination	231	72.2	225	70.3
Probability ≥ 50% of contamination	261	81.6	258	80.6	Probability ≥ 50% of contamination	254	79.4	248	77.8
100% of contamination	315	98.4	314	98.1	100% of contamination	311	97.1	308	96.2

This table shows the number of SGC community members (as an absolute and as a proportion of study group [*n* = 320]) who said they would participate in anti-helminthic MDA treatment programs or allow a family member to participate in anti-helminthic MDA treatment programs targeting mansonellosis or STH infections. Data for MDAs conducted in endemic regions without prior prevalences surveys and data for MDAs for which the community member responding believes there is ≥50% they are infected with the targeted helminth is shown.

**Table 6:** Significant differences in community support for different types of MDA treatment regimens for helminthic diseases in SGC.

	STH		Mansonellosis	
Infection Status	*p*-Value	*p*-Value (Family Member)	*p*-Value	*p*-Value (Family Member)
**100% vs. 50%**	0.00001	0.00001	0.00001	0.00001
**50% vs. unknown**	0.006425	0.007195	0.033823	0.038426

This table highlights significant differences in community support for helminth infection MDA programs. Quoted *p*-values were obtained from chi-squared tests that compared the number of SGC community members who said they would participate in a given MDA program with the number of SGC community members that said they would participate in an equivalent program where known prevalence levels were different.

## Data Availability

All the data used for this study is presented in the results and Supplementary Material of this manuscript. Access to raw questionnaire survey data will be provided by the corresponding author to all who make reasonable requests.

## Supplementary Materials

The following supporting information can be downloaded at: https://www.mdpi.com/article/10.3390/tropicalmed10070186/s1; Figure S1: project questionnaire; Figure S2: informed consent form; Figure S3: mansonellosis fact sheet; Figure S4: soil transmitted helminths fact sheet.
