# Supplementary material for: High Levels of Community Support for Mansonellosis Interventions in an Endemic Area of the Brazilian Amazon
Source: Trop Med Infect Dis. 2025 Jul 2;10(7):186. doi: 10.3390/tropicalmed10070186 (PMC12300409; doi:10.3390/tropicalmed10070186)
Supplement: Supplementary file 1 [file tropicalmed-10-00186-s001.zip › tropicalmed-3696368-supplementary.pdf]

Figure S1

APÊNDICE A – INTRUMENTO DE PESQUISA

QUESTIONÁRIO DE PESQUISA

TÍTULO DA PESQUISA: AVALIAÇÃO DO APOIO COMUNITÁRIO PARA ADMINISTRAÇÃO EM MASSA DE MEDICAMENTOS ANTI-HELMÍNTICOS NO MUNICÍPIO DE SÃO GABRIEL DA CACHOEIRA, AMAZONAS

DADOS DE IDENTIFICAÇÃO

Nome:		Registro:
Bairro:	Rua:	Nº
Ponto de referência:		
Data da entrevista:		

PARTE A – VARIÁVEIS SOCIODEMOGRÁFICAS

1. Idade__________ anos
2. Sexo ☐M ☐ F
3. Cor ☐ branca ☐ preta ☐ parda ☐ outros. Especifique:____________________________
4. trabalho/ocupação:
5. Local de trabalho:
6. Estado civil ☐ solteiro ☐ casado ☐ viúvo ☐ divorciado ☐ outros. Especifique:_______________
7. Grau de instrução:<br>☐Analfabeto / Fundamental I incompleto<br>☐Fundamental I completo / Fundamental II incompleto<br>☐Fundamental completo/Médio incompleto<br>☐Médio completo/Superior incompleto<br>☐Superior completo
8. Renda familiar:<br>☐ Menor que 3 salários mínimos<br>☐ Entre 3 e 6 salários mínimos<br>☐ Entre 6 e 10 salários mínimos<br>☐ Acima de 10 salários mínimos<br>☐ Não sabe referir
9. Local de residência (Perímetro) ☐ urbano ☐ rural
10. Número de pessoas que residem na mesma casa:____________

Figure S1

11. Há quanto tempo reside nesse endereço? _____________

12. situação econômica		QUANTIDADE QUE POSSUI			
**ITENS DE CONFORTO**	**NÃO POSSUI**	**1**	**2**	**3**	**4+**
Banheiros					
Empregados domésticos					
Automóveis					
Microcomputador					
Lava-louça					
Geladeira					
Freezer					
Lava-roupa					
DVD					
Micro-ondas					
Motocicleta					
Secadora de roupa					

**Água utilizada no domicílio é proveniente:**	
1	Rede geral de distribuição
2	Poço ou nascente
3	Outro meio
**Considerando o trecho do domicílio, a rua é:**	
1	Asfaltada/pavimentada
2	Terra/cascalho
**Grau de instrução do chefe da família** (a pessoa que contribui com a maior parte de renda do domicílio):	
	Analfabeto / Fundamental I incompleto
	Fundamental I completo / Fundamental II incompleto
	Fundamental completo/Médio incompleto
	Médio completo/Superior incompleto
	Superior completo

Classificação segundo ABEP, 2021.

PARTE B - CONHECIMENTO SOBRE DOENÇAS HELMÍNTICAS REGIONAIS E MÉTODOS DE CONTROLE

1. **Você já ouviu falar em alguma doença causada por vermes (Helmintos Transmitidos pelo Solo)? Se SIM, quais?**
   ☐ SIM: ______________________________________ ☐ NÃO
2. **Você já ouviu falar em algum desses vermes ou doenças?**

- Amarelão (ancilostomíase)☐ SIM ☐ NÃO
- Ameba (Amebíase) ☐ SIM ☐ NÃO
- Lombriga (ascaridíase) ☐ SIM ☐ NÃO
- Solitária (Cisticercose) ☐ SIM ☐ NÃO

- Tuxina (Oxiurose) ☐ SIM ☐ NÃO

3. **Você já ouviu falar em Filariose/filária (mansonelose)?**
   ☐ SIM ☐ NÃO
4. **Você já ouviu falar em pira (sarna)?**
   ☐ SIM ☐ NÃO
5. **Você já ouviu falar em um tipo de conjuntivite chamado tracoma?**
   ☐ SIM ☐ NÃO
6. **Você sabia que a contaminação por vermes (Helmintos Transmitidos pelo Solo) é muito comum aqui na sua região?**
   ☐ SIM ☐ NÃO
7. **Você sabia que a contaminação por Filariose/filária (mansonelose) é muito comum aqui na sua região?**
   ☐ SIM ☐ NÃO
8. **Você sabia que a contaminação por pira (sarna) e tracoma (conjuntivite) é muito comum aqui na sua região?**
   ☐ SIM ☐ NÃO
9. **Você sabia que usar sapatos ou sandálias poderia protegê-lo contra contaminação por vermes (Helmintos Transmitidos pelo Solo)?**
   ☐ SIM ☐ NÃO
10. **Você sabia que lavar as mãos, "verduras" e beber água limpa (de garrafão/tratada/filtrada) poderia protegê-lo contra contaminação por vermes (Helmintos Transmitidos pelo Solo)?**
    ☐ SIM ☐ NÃO
11. **Você sabia que as verminoses (Helmintíases Transmitidas pelo Solo) poderiam ser curadas de maneira rápida e facilmente com remédios?**
    ☐ SIM ☐ NÃO
12. **Você conhece algum remédio usado para tratar vermes (Helmintos Transmitidos pelo Solo)? Se SIM, quais?**
    ☐ SIM: ______________________________ ☐ NÃO
13. **Você conhece algum remédio usado para tratar Filariose/filária (mansonelose)? Se SIM, quais?**
    ☐ SIM: ______________________________ ☐ NÃO
14. **Você conhece algum remédio usado para tratar pira (sarna) e tracoma (conjuntivite)? Se SIM, quais?**
    ☐ SIM: ______________________________ ☐ NÃO

15. **Você conhece algum desses remédios (medicamentos)?**

- Albendazol ☐ SIM ☐ NÃO
- Azitromicina ☐ SIM ☐ NÃO
- Dietilcarbamazina (DEC) ☐ SIM ☐ NÃO
- Doxiciclina ☐ SIM ☐ NÃO
- Ivermectina ☐ SIM ☐ NÃO
- Mebendazol ☐ SIM ☐ NÃO
- Praziquantel ☐ SIM ☐ NÃO

Figure S1

16. Você já tomou algum desses remédios (medicamentos)?

- Albendazol ☐ SIM ☐ NÃO
- Azitromicina ☐ SIM ☐ NÃO
- Dietilcarbamazina (DEC) ☐ SIM ☐ NÃO
- Doxiciclina ☐ SIM ☐ NÃO
- Ivermectina ☐ SIM ☐ NÃO
- Mebendazol ☐ SIM ☐ NÃO
- Praziquantel ☐ SIM ☐ NÃO

Se SIM, para tratar/controlar qual doença? ________________________________

PARTE C - EXPERIÊNCIA COM DOENÇAS HELMÍNTICAS REGIONAIS

17. Você já teve alguma dessas doenças?

- Amarelão (ancilostomíase)☐ SIM ☐ NÃO ☐ NÃO SEI
- Ameba (amebíase) ☐ SIM ☐ NÃO ☐ NÃO SEI
- Filariose/filária (mansonelose) ☐ SIM ☐ NÃO ☐ NÃO SEI
- Lombriga (ascaridíase) ☐ SIM ☐ NÃO ☐ NÃO SEI
- Pira (sarna) ☐ SIM ☐ NÃO ☐ NÃO SEI
- Solitária (Cisticercose) ☐ SIM ☐ NÃO ☐ NÃO SEI
- Tracoma (tipo de conjuntivite) ☐ SIM ☐ NÃO ☐ NÃO SEI
- Tuxina (Oxiurose) ☐ SIM ☐ NÃO ☐ NÃO SEI

Outras verminoses:____

18. Alguém da sua família já teve alguma dessas doenças?

- Amarelão (ancilostomíase)☐ SIM ☐ NÃO ☐ NÃO SEI
- Ameba (amebíase) ☐ SIM ☐ NÃO ☐ NÃO SEI
- Filariose/filária (mansonelose) ☐ SIM ☐ NÃO ☐ NÃO SEI
- Lombriga (ascaridíase) ☐ SIM ☐ NÃO ☐ NÃO SEI
- Pira (sarna) ☐ SIM ☐ NÃO ☐ NÃO SEI
- Solitária (Cisticercose) ☐ SIM ☐ NÃO ☐ NÃO SEI
- Tracoma (tipo de conjuntivite) ☐ SIM ☐ NÃO ☐ NÃO SEI
- Tuxina (Oxiurose) ☐ SIM ☐ NÃO ☐ NÃO SEI

Outras verminoses:____

PARTE D - ATITUDES EM RELAÇÃO AOS TRATAMENTOS DE DOENÇAS HELMÍNTICAS REGIONAIS

19. Você tomaria um remédio (medicamento) por via oral para tratar vermes (Helmintos Transmitidos pelo Solo)?

☐ SIM ☐ NÃO

Se SIM você tomaria por:

- 1 dia ☐ SIM ☐ NÃO

Figure S1

- 2 dias ☐ SIM ☐ NÃO
- 3 dias ☐ SIM ☐ NÃO
- 7 dias ☐ SIM ☐ NÃO
- 4 semanas ☐ SIM ☐ NÃO

20. **Você permitiria que alguém da sua família por quem você fosse responsável (filho, por exemplo) tomasse um remédio (medicamento) por via oral para tratar vermes (Helmintos Transmitidos pelo Solo)?**

    ☐ SIM ☐ NÃO

    Se SIM você permitiria por:

- 1 dia ☐ SIM ☐ NÃO
- 2 dias ☐ SIM ☐ NÃO
- 3 dias ☐ SIM ☐ NÃO
- 7 dias ☐ SIM ☐ NÃO
- 4 semanas ☐ SIM ☐ NÃO

21. **Você participaria de um programa (campanha) de controle de vermes (Helmintos Transmitidos pelo Solo) realizado em toda a sua comunidade no qual você tivesse que tomar (por via oral) um remédio (medicamento) (em dose única) mesmo sem saber se você estava contaminado?**

    ☐ SIM ☐ NÃO

22. **Você permitiria que alguém da sua família (seu filho, por exemplo) participasse de um programa (campanha) de controle de vermes (Helmintos Transmitidos pelo Solo) realizado em toda a sua comunidade no qual ele tivesse que tomar (por via oral) um remédio (medicamento) (em dose única) mesmo sem saber se ele estava contaminado?**

    ☐ SIM ☐ NÃO

23. **Você participaria de um programa (campanha) de controle de vermes (Helmintos Transmitidos pelo Solo) realizado em toda a sua comunidade no qual você tivesse que tomar (por via oral) um remédio (medicamento) (em dose única) se existisse uma possibilidade maior ou igual a 50% de você estar contaminado?**

    ☐ SIM ☐ NÃO

24. **Você permitiria que alguém da sua família (seu filho, por exemplo) participasse de um programa (campanha) de controle de vermes realizado em toda a sua comunidade no qual ele tivesse que tomar (por via oral) um remédio (medicamento) (em dose única) se existisse uma possibilidade maior ou igual a 50% dele estar contaminado com vermes (Helmintos Transmitidos pelo Solo)?**

    ☐ SIM ☐ NÃO

25. **Você tomaria um remédio (medicamento) por via oral para controlar filariose/filária (mansonelose)?**

    ☐ SIM ☐ NÃO

    Se SIM você tomaria por:

- 1 dia ☐ SIM ☐ NÃO
- 2 dias ☐ SIM ☐ NÃO
- 3 dias ☐ SIM ☐ NÃO
- 7 dias ☐ SIM ☐ NÃO
- 4 semanas ☐ SIM ☐ NÃO

Figure S1

26. Você permitiria que alguém da sua família por quem você fosse responsável (filho, por exemplo) tomasse um remédio (medicamento) por via oral para controlar filariose/filária (mansonelose)?

☐ SIM ☐ NÃO

Se SIM você permitiria por:

- 1 dia ☐ SIM ☐ NÃO
- 2 dias ☐ SIM ☐ NÃO
- 3 dias ☐ SIM ☐ NÃO
- 7 dias ☐ SIM ☐ NÃO
- 4 semanas ☐ SIM ☐ NÃO

27. Você participaria de um programa (campanha) de controle de filariose/filária (mansonelose) realizado em toda a sua comunidade no qual você tivesse que tomar (por via oral) um remédio (medicamento) (em dose única) mesmo sem saber se você estava contaminado?

☐ SIM ☐ NÃO

28. Você permitiria que alguém da sua família (seu filho, por exemplo) participasse de um programa (campanha) de controle de filariose/filária (mansonelose) realizado em toda a sua comunidade no qual ele tivesse que tomar (por via oral) um remédio (medicamento) (em dose única) mesmo sem saber se ele estava contaminado?

☐ SIM ☐ NÃO

29. Você participaria de um programa (campanha) de controle de filariose/filária (mansonelose) realizado em toda a sua comunidade no qual você tivesse que tomar (por via oral) um remédio (medicamento) (em dose única) se existisse uma possibilidade maior ou igual a 50% de você estar contaminado?

☐ SIM ☐ NÃO

30. Você permitiria que alguém da sua família (seu filho, por exemplo) participasse de um programa (campanha) de controle de filariose/filária (mansonelose) realizado em toda a sua comunidade no qual ele tivesse que tomar (por via oral) um remédio (medicamento) (em dose única) se existisse uma possibilidade maior ou igual a 50% dele estar contaminado?

☐ SIM ☐ NÃO

31. Você tomaria um remédio (medicamento) por via oral para tratar pira (sarna) ou tracoma (tipo de conjuntivite)?

☐ SIM ☐ NÃO

Se SIM você tomaria por:

- 1 dia ☐ SIM ☐ NÃO
- 2 dias ☐ SIM ☐ NÃO
- 3 dias ☐ SIM ☐ NÃO
- 7 dias ☐ SIM ☐ NÃO
- 4 semanas ☐ SIM ☐ NÃO

32. Você permitiria que alguém da sua família por quem você fosse responsável (filho, por exemplo) tomasse um remédio (medicamento) por via oral para para tratar pira (sarna) ou tracoma (tipo de conjuntivite)?

☐ SIM ☐ NÃO

Se SIM você permitiria por:

- 1 dia ☐ SIM ☐ NÃO
- 2 dias ☐ SIM ☐ NÃO
- 3 dias ☐ SIM ☐ NÃO
- 7 dias ☐ SIM ☐ NÃO
- 4 semanas ☐ SIM ☐ NÃO

33. **Você participaria de um programa (campanha) de controle de pira (sarna) ou tracoma (tipo de conjuntivite) realizado em toda a sua comunidade no qual você tivesse que tomar (por via oral) um remédio (medicamento) (em dose única) mesmo sem saber se você estava contaminado?**
    ☐ SIM ☐ NÃO
34. **Você permitiria que alguém da sua família (seu filho, por exemplo) participasse de um programa (campanha) de controle de pira (sarna) ou tracoma (tipo de conjuntivite) realizado em toda a sua comunidade no qual ele tivesse que tomar (por via oral) um remédio (medicamento) (em dose única) mesmo sem saber se ele estava contaminado?**
    ☐ SIM ☐ NÃO
35. **Você participaria de um programa (campanha) de controle de pira (sarna) ou tracoma (tipo de conjuntivite) realizado em toda a sua comunidade no qual você tivesse que tomar (por via oral) um remédio (medicamento) (em dose única) se existisse uma possibilidade maior ou igual a 50% de você estar contaminado?**
    ☐ SIM ☐ NÃO
36. **Você permitiria que alguém da sua família (seu filho, por exemplo) participasse de um programa (campanha) de controle de pira (sarna) ou tracoma (tipo de conjuntivite) realizado em toda a sua comunidade no qual ele tivesse que tomar (por via oral) um remédio (medicamento) (em dose única) se existisse uma possibilidade maior ou igual a 50% dele estar contaminado?**
    ☐ SIM ☐ NÃO

Figure S2

APÊNDICE C – TCLE

TERMO DE CONSENTIMENTO LIVRE E ESCLARECIDO (TCLE)

O(A) Sr(a) está sendo convidado(a) a participar do projeto de pesquisa: **Avaliação do Apoio Comunitário para Administração em Massa de Medicamentos Anti-helmínticos no Município de São Gabriel da Cachoeira, Amazonas**, cuja pesquisadora responsável é **Carla Letícia Gomes Simão,** discente de Mestrado em Saúde Pública do Instituto Leônidas & Maria Deane da Fundação Oswaldo Cruz (ILMD/FIOCRUZ), sob orientação do Prof. Dr. James Lee Crainey e da Profª Drª Marilaine Martins**.** Nesta pesquisa, queremos saber sobre o seu conhecimento, e o da sua comunidade, sobre os tipos de remédios (medicamentos) contra a filária (Mansonelose). Além disso, também queremos saber se vocês aceitariam esses remédios como forma de controle da filária (programas que tratam ao mesmo tempo toda a comunidade). Como muitos dos remédios usados para tratar a filária, eles também tratam outros vermes (Helmintos Transmitidos pelo Solo) e são usados contra a sarna (pira) e Tracoma (um tipo de conjuntivite). Também queremos saber sobre o entendimento das pessoas sobre os efeitos desses remédios, e se isso afetaria o seu apoio no tratamento da filária. Esse convite se deve ao fato de o(a) Sr(a) ser morador (a) da área urbana do município de São Gabriel da Cachoeira.

O(A) Sr(a) tem liberdade de recusar em participar ou retirar seu consentimento, em qualquer fase da pesquisa, sem penalização alguma.

Caso aceite a sua participação será de responder perguntas de um questionário à pesquisadora do projeto. O tempo de duração da aplicação do questionário será de aproximadamente trinta minutos.

A qualquer momento, durante a pesquisa, ou posteriormente, o(a) Sr(a) poderá solicitar da pesquisadora informações sobre sua participação e/ou sobre a pesquisa, o que poderá ser feito através dos meios de contato presentes neste Termo.

O questionário será armazenado, em arquivos de computadores, mas somente terão acesso as informações a pesquisadora e seus orientadores. Ao final da pesquisa, todo material será mantido em arquivo por pelo menos cinco anos e com no final deste prazo, se houver necessidade de mais tempo, nós voltaremos pedir a sua autorização para continuar utilizando as informações que o(a) Sr(a) está nos fornecendo e/ou essas informações serão descartadas.

O(A) Sr(a) não será identificado (a) em nenhuma publicação. Não há riscos de exposição de sua imagem, o seu nome não será mencionado em publicações ou relatórios produzidos para este estudo.

Toda pesquisa com seres humanos envolve riscos aos participantes. Nesta pesquisa os riscos para o(a) Sr.(a) são: Ficar cansado ou entediado ao responder o questionário, com o tempo de aplicação estimado em 30 minutos no máximo. Poderá ainda apresentar um leve desconforto ao responder a qualquer um dos itens durante a entrevista. Para diminuir esses riscos, o(a) Sr(a) poderá interromper momentaneamente as respostas, e poderá retomá-las a qualquer momento do ponto onde parou. Como benefício da sua participação o(a) Sr(a) receberá informações sobre a transmissão e tratamento das verminoses, doenças transmitidas por bactérias, além disso, projetos e programas de controle da filária (mansonelose) na região poderão ser realizados.

Se julgar necessário, o(a) Sr(a) dispõe de tempo para que possa pensar sobre sua participação, consultando, se necessário, seus familiares ou outras pessoas que possam ajudá-los na tomada de decisão livre e esclarecida.

Figure S2

Para participar desta pesquisa, o(a) Sr(a) não terá nenhum custo, nem receberá qualquer vantagem financeira. Diante de eventuais danos, identificados e comprovados, causados pela pesquisa, o(a) Sr(a) tem assegurado o direito à indenização.

Asseguramos ao(à) Sr(a) o direito de assistência integral gratuita devido a danos diretos/indiretos e imediatos/tardios decorrentes da participação no estudo ao participante, pelo tempo que for necessário.

Garantimos ao(à) Sr(a) a manutenção do segredo (sigilo e da privacidade) de sua participação e de seus dados durante todas as fases da pesquisa e posteriormente na divulgação científica.

O(A) Sr(a) pode entrar com contato com a pesquisadora responsável **Carla Letícia Gomes Simão** a qualquer tempo para informações adicionais no endereço: Rua Teresina, 476 – Adrianópolis – CEP – 69057-070, Manaus-AM. Telefone: (92) 3221-2323. E-Mail: carlalgsimao@gmail.com.

Em caso de dúvida quanto à condução ética do estudo, entre em contato com o Comitê de Ética em Pesquisa da Fundação de Medicina Tropical Doutor Heitor Vieira Dourado (CEP/FMT-HVD), localizado Av. Pedro Teixeira, 25 – Dom Pedro I - Manaus – AM - CEP: 69040-000. Telefone: (92) 2127-3572. E-Mail: cep@fmt.am.gov.br. O CEP/FMT-HVD é um colegiado multi e transdisciplinar, independente, criado para defender os interesses dos participantes da pesquisa em sua integridade e dignidade e para contribuir no desenvolvimento da pesquisa dentro de padrões éticos.

Este documento (TCLE) será elaborado em duas VIAS, que serão rubricadas em todas as suas páginas, exceto a com as assinaturas, e assinadas ao seu término pelo(a) Sr(a) e pela pesquisadora responsável, ficando uma via com cada um.

CONSENTIMENTO PÓS-INFORMAÇÃO
Li e concordo em participar da pesquisa.

São Gabriel da Cachoeira, ____/_____/_____

____

Assinatura do Participante
Nome do Participante:

____

Assinatura do Pesquisador Responsável
Nome do Pesquisador:

IMPRESSÃ

Figure S3

APÊNDICE B – FOLHETO INFORMATIVO SOBRE HELMINTÍASES

Helmintos Transmitidos pelo Solo (HTSs) da Amazônia

Ciclo de transmissão de *Strongyloides stercoralis*

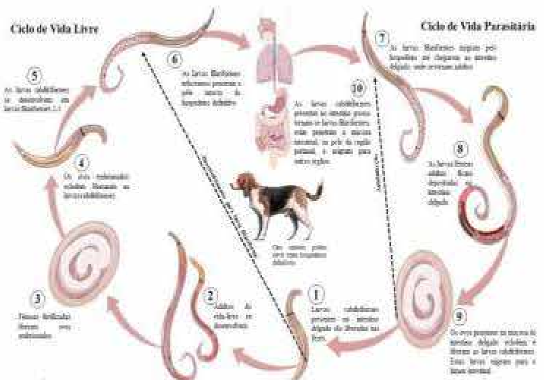

Figura 1

As infecções por Helmintos Transmitidos pelo Solo (HTS) são muito comuns na região amazônica brasileira e são contraídas através do contato com solo contaminado. Na Amazônia Brasileira os parasitas HTS mais importantes são: *Ascaris lumbricoides*, *Trichuris trichiura*, *Strongyloides stercoralis* e *Necator americanos*. Na Amazônia Brasileira e demais localidades, os HTS causam uma carga de doença muito significativa para muitas pessoas de baixa renda. Nesta região, as pessoas são commumente infectadas com *Strongyloides stercoralis* e *Necator americanus* andando descalço sobre o solo contaminado e são commumente infectados com *Ascaris lumbricoides* e *Trichuris trichiura* através do consumo de água ou alimentos contaminados. O uso de sapatos ou sandálias pode efetivamente proteger contra infecções por *Strongyloides stercoralis* e *Necator americanus*, e o hábito de lavar as mãos e vegetais, além de beber água potável (envasada/tratada/filtrada) podem proteger contra infecções por *Ascaris lumbricoides* e *Trichuris trichiura*. Garantir o livre acesso a sistemas adequados de gerenciamento de banheiros e esgoto para toda a comunidade é uma forma eficaz de proteção contra todos esses parasitas. Para ver os diferentes modos pelos quais as pessoas são infectadas e/ou infectam outras pessoas com HTS observe os ciclos de vida dos parasitas causadores (figura 1 a 4).

Ciclo de transmissão de *Ascaris lumbricoides*

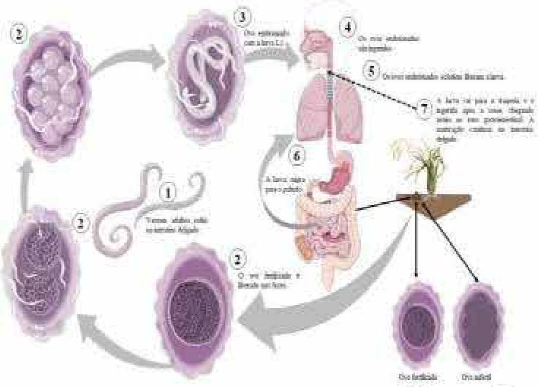

Figura 3

Ciclo de transmissão de *Necator americanus*

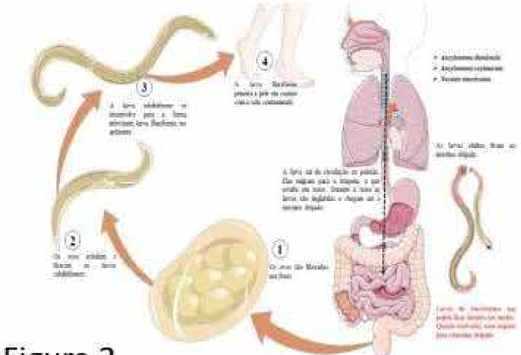

Figura 2

Tratamento de HTSs

Todos os HTS que ocorrem na região amazônica podem ser curados com tratamentos medicamentosos simples, os quais têm poucos ou nenhum efeito colateral. Três dias de tratamento com 400 mg de albendazol ou 500 mg de mebendazol podem curar infecções por *Ascaris lumbricoides*, *Trichuris trichiura* e *Necator americanus*. Da mesma forma, tratamentos de três dias com 200 µg/kg de ivermectina podem curar infecções por *Ascaris lumbricoides*, *Trichuris trichiura* e *Strongyloides stercoralis*. Espera-se que um programa de controle da mansonelose que alterne a cada seis meses entre tratamentos de três dias com ivermectina e tratamentos de três dias com mebendazol tenha um impacto muito significativo na ocorrência de HTS e mansonelose na região, caso seja sustentado por 15 anos ou mais. Programas de controle da mansonelose também poderiam efetivamente utilizar tratamentos de dose única com essas drogas, embora tais programas não fossem tão eficazes contra infecções por *Trichuris trichiura* e *Strongyloides stercoralis*.

Ciclo de transmissão de *Trichuris trichiura*

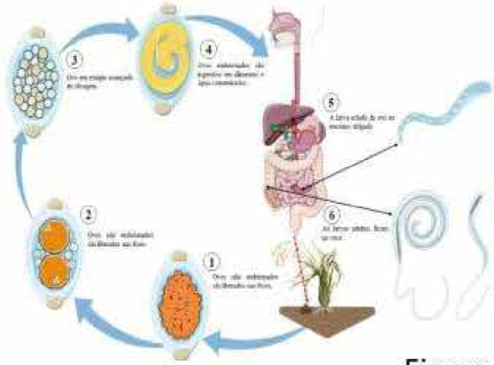

Figura 4

Ministério da Saúde
FIOCRUZ
Fundação Oswaldo Cruz
ILMD
Instituto Leônidas & Maria Deane
Fiocruz Amazônia

Figure S4

Mansonelose: uma doença crônica infecciosa da região Amazônica

Ministério da Saúde
FIOCRUZ
Fundação Oswaldo Cruz
ILMD

Folheto informativo sobre mansonelose

A mansonelose é uma doença crônica causada por parasitas filariais que vivem e se reproduzem dentro do corpo humano. A maioria das pessoas infectadas permanece infectada durante toda a vida adulta e muitas nunca saberão que estão infectadas. Os parasitas são muito comuns em quase todos os municípios do interior do estado do Amazonas e em alguns estima-se que mais da metade dos moradores estejam infectados. Embora ainda não conheçamos todos os danos que as infecções por mansonelose causam às pessoas, sabemos que podem causar lesões na córnea ocular, bem como alguns sintomas leves, como dores de cabeça. As pessoas são infectadas quando são picadas por insetos vetores infectados (piuns ou maruins). Atualmente, não há programas coordenados de controle da mansonelose na Amazônia Brasileira ou em qualquer outro lugar, embora existam dois métodos viáveis baseados em medicamentos que poderiam controlar a doença.

Controle da mansonelose com anti-helmínticos tradicionais

A organização mundial de saúde (OMS) utiliza medicamentos anti-helmínticos tradicionais (incluindo a ivermectina) para eliminar as microfilárias do sangue e dos tecidos da pele de indivíduos infectados (ver figura) para que os insetos vetores (piuns e maruins) não sejam infectados e transmitam o parasita. Os medicamentos são administrados por via oral em todos os indivíduos que concordam em participar em determinada área endêmica escolhida, sem primeiro verificar se o participante está infectado com o parasita-alvo. Esses tratamentos medicamentosos interrompem a transmissão, mas não matam os parasitas filariais adultos, o que significa que precisam ser repetidos anualmente até que os parasitas adultos morram naturalmente (após cerca de 15 a 20 anos). Não há nenhum benefício clínico imediato claro para os participantes desses tipos de programas e uma proporção muito pequena das pessoas que recebem um tratamento medicamentoso anti-helmíntico tradicional sofre de náusea, diarreia e/ou vômito. No entanto, quando uma grande proporção da comunidade onde a doença é endêmica concorda em participar, há benefícios para a saúde de todos da região. Os programas da OMS que usaram essas abordagens aliviaram muito o sofrimento e eliminaram os focos de oncocercose em toda a América Latina. Programas semelhante poderiam ser usados para controlar ou eliminar a mansonelose aqui na Amazônia Brasileira. Participantes dos programas de controle da mansonelose baseados em medicamentos anti-helmínticos tradicionais poderiam também obter benefícios adicionais à saúde, uma vez que esses tratamentos para mansonelose também os curariam de infecções por Helmintos Transmitidos pelo Solo (HTS), como *Ascaris lumbricoides*, *Trichuris trichiura*, *Strongyloides stercoralis* e *Necator americanos*.

Ciclo de transmissão da mansonelose

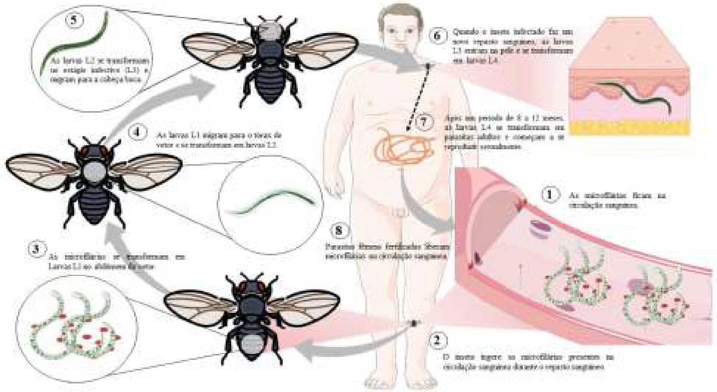

Controle da mansonelose com antibióticos

As infecções por mansonelose, tal como muitas infecções filariais humanas, podem ser curadas através de tratamentos com antibióticos. Esses tratamentos funcionam matando uma bactéria simbiótica que vive dentro das células dos parasitas filarias e até agora eram feitos apenas com o antibiótico doxiciclina. Quando a OMS utiliza tratamentos com doxiciclina para controle das doenças filarias, a droga é administrada diariamente por 4 semanas e apenas em pessoas que tiveram suas infecções confirmadas. Os longos cursos de tratamento necessários para que a doxiciclina funcione tornam seu uso muito mais exigente logisticamente e financeiramente do que os programas que usam drogas anti-helmínticas tradicionais e, portanto, os antibióticos não são amplamente utilizados para o controle das doenças filariais. No entanto, novos programas de tratamento com antibióticos nos quais os participantes são curados de suas infecções após um curto tratamento de 1 semana com esses medicamentos podem ser lançados na Amazônia Brasileira nos próximos 10 anos e tratamentos curativos de dose única podem estar disponíveis em 20 anos. Assim como os tratamentos anti-helmínticos tradicionais para mansonelose, os tratamentos à base de antibióticos podem proporcionar aos indivíduos benefícios à saúde além da cura de suas infecções por mansonelose. Por exemplo, infecções por tracoma, sarna e bouba poderiam ser potencialmente curadas por tratamentos com antibióticos usados para tratar infecções por mansonelose. Da mesma forma, alguns tratamentos com antibióticos podem ser usados com segurança em combinação com anti-helmínticos tradicionais, o que significa que os programas de controle da mansonelose podem fornecer amplos benefícios à saúde dos participantes.
